# Real-time monitoring of infant theta power during naturalistic social experiences

**DOI:** 10.1016/j.dcn.2023.101300

**Published:** 2023-09-21

**Authors:** Elena Throm, Anna Gui, Rianne Haartsen, Pedro F. da Costa, Robert Leech, Emily J.H. Jones

**Affiliations:** a Centre for Brain and Cognitive Development, Department of Psychological Science, Birkbeck, University of London, Henry Wellcome Building, Malet Street, London WC1E 7HX, United Kingdom; b Centre for Brain and Cognitive Development, Department of Psychological Science, Birkbeck, University of London, TodderLab, Malet Street, London WC1E 7HX, United Kingdom; c Department of Neuroimaging, Institute of Psychiatry, Psychology and Neuroscience, King’s College London, de Crespigny Road, London SE5 8AB, United Kingdom

**Keywords:** EEG theta oscillation, Social attention, Individual differences, Real-time analysis, Infant, Naturalistic context

## Abstract

Infant-directed speech and direct gaze are important social cues that shape infant’s attention to their parents. Traditional methods for probing their effect on infant attention involve a small number of pre-selected screen-based stimuli, which do not capture the complexity of real-world interactions. Here, we used neuroadaptive Bayesian Optimization (NBO) to search a large ‘space’ of different naturalistic social experiences that systematically varied in their visual (gaze direct to averted) and auditory properties (infant directed speech to nonvocal sounds). We measured oscillatory brain responses (relative theta power) during episodes of naturalistic social experiences in 57 typically developing 6- to 12-month-old infants. Relative theta power was used as input to the NBO algorithm to identify the naturalistic social context that maximally elicited attention in each individual infant. Results showed that individual infants were heterogeneous in the stimulus that elicited maximal theta with no overall stronger attention for direct gaze or infant-directed speech; however, individual differences in attention towards averted gaze were related to interpersonal skills and greater likelihood of preferring speech and direct gaze was observed in infants whose parents showed more positive affect. Our work indicates NBO may be a fruitful method for probing the role of distinct social cues in eliciting attention in naturalistic social contexts at the individual level.

## 1 Introduction

Young infants spend a substantial proportion of their waking hours with their parents or caregivers. These social experiences are thought to contribute to the progressive specialisation of brain regions in the ‘social brain network’ and support the emerging complexity of social behaviour over the first year of life (Johnson, 2011). Attention to people yields opportunities to learn to recognise other people, interpret their speech and learn about their actions on the world (Carnevali et al., 2022). Preferences for social stimuli like faces and voices are present at birth or even prenatally and may ensure the infant is drawn to important sources of information and care in their environment (Shultz et al., 2018). Identifying the specific social cues that maximally capture infant attention is important to testing the range of theoretical frameworks put forward to explain social development and understand the emergence of social expertise in infancy.

Certain social cues have been proposed to be particularly important in modulating attention from early infancy. First, direct gaze may signal to an infant that an interactive partner is attending to them or addressing them. For example, the natural pedagogy theory *(*Csibra and Gergely, 2009, Csibra and Gergely, 2011) proposes that direct gaze, infant-directed speech and contingency are ostensive or communicative cues that signal to the infant that an interaction partner is addressing them in order to convey knowledge, and to which infants have an innate tendency to react (Senju and Csibra, 2008). Indeed, direct gaze elicits differential responses to averted gaze from birth (Farroni et al., 2006, Farroni et al., 2002, Farroni et al., 2000). Infants are also more likely to recognise faces when accompanied by direct eye gaze (Rigato et al., 2011). Gaze direction can also direct infant attention; by 8 months, most infants can use gaze to follow the direction of attention of their social partner (Senju and Csibra, 2008, del Bianco et al., 2018, Szufnarowska et al., 2014). Attention to gaze may be initially subcortically-mediated, before becoming cortically controlled in later infancy (Johnson et al., 2015, Senju et al., 2008). In summary, gaze is thought to be an important feature in capturing and directing early infant attention.

In addition to visual cues, auditory cues are also important. Infant-directed speech (IDS) is the way adults adapt their speech when talking to a baby, and differs in numerous features from adult-directed speech, including higher and more variable pitch, longer pauses, better articulation (Cooper and Aslin, 1990), larger lip movements (Green et al., 2010), stronger rhythmic synchronisation and higher temporal regularity (Leong et al., 2017a). Pre-recorded and presented in isolation, IDS has modulated correlates of infant attention (Frank et al., 2020, Menn et al., 2022, Lopera-Perez et al., 2022), including theta power (Zhang et al., 2011), possibly due to entrainment between neural oscillations and speech (Nencheva and Lew-Williams, 2022). In optical and functional imaging studies, 5-month-old infants showed stronger brain responses associated with social attention in response to infant-directed speech compared to nonvocal sounds (e.g., toys) (Blasi et al., 2011, Lloyd-Fox et al., 2013). The combination of live auditory (IDS vs ADS) and visual (direct vs averted) ostensive cues may be particularly powerful in eliciting social brain responses in early infancy (Lloyd-Fox et al., 2015). Thus, infant-directed speech is thought to be another important factor in capturing attention towards people in early development.

Most of the literature showing important roles for direct gaze and infant-directed speech in eliciting strong brain engagement has employed traditional paradigms in which infants respond to isolated stimuli presented on a screen, which are preselected by the researchers and are presented following a set stimulus-presentation schedule. However, in real life, cues are embedded in complex, dynamic environments and the infant selects their own experiences of interest. Studies using observational methods have increasingly challenged the generalisability of screen-based studies to real-world contexts (e.g (Wass, 2014).). Further, individual differences are likely critical in determining which features of social interaction are most optimal, based on the infant-specific interplay between behaviour, attention, and the brain (Nencheva and Lew-Williams, 2022). Determining the stimuli that maximally elicit the activation of particular neural systems at the level of the individual infant and in more naturalistic settings requires new experimental paradigms. Our goal was to develop an approach that combined theory-informed experimental design with naturalistic experiences (Wass and Jones, 2023).

Neuroadaptive Bayesian Optimization (NBO) is a novel experimental approach that flips the traditional design by moving away from studying a group’s averaged response to a few stimuli towards an individual’s response to various stimuli. NBO has proven successful in identifying the stimulus that is more likely to maximise a target brain response (optimum) in adults (Lorenz et al., 2017, Lorenz et al., 2018, Lorenz et al., 2016) and infants (da Costa et al., 2021). NBO identifies the infant’s preferred cues from a broad range of options by directly comparing individual infants’ brain responses to different elements of a naturalistic social context varied within an experimental search space. By adapting the stimulus presentation according to the individual’s response, NBO allows us to study the response to experiences generated by the individual infant. Here, we apply NBO to examine how infant social attention is modulated by gaze and auditory input during live social experiences.

To capture attentive brain response to social cues, we used Electroencephalography (EEG). EEG is a useful technique to study infant neural responses in naturalistic settings due to its non-invasiveness and portability. Synchronised activity in the theta band of the EEG signal (theta power) measured over frontal regions of the brain has been related to attentive brain states (Xie et al., 2018) and has been proposed as an index of attention and active learning (Begus and Bonawitz, 2020). Further, theta power has been shown to differentiate processing of social from nonsocial stimuli in infancy. For example, a stronger increase of theta power between 6 and 12 months was observed for social compared to nonsocial videos (Jones et al., 2017, Jones et al., 2020, Haartsen et al., 2022). In a naturalistic context, elevated theta power towards social vs nonsocial conditions was observed at 6 months, and this differential response showed an even greater increase in strength and spatial extent over the second half of the first year of life (Jones et al., 2015). However, while there is strong evidence for elevated theta power during social vs nonsocial stimulation, particularly in a naturalistic live context compared to pre-recorded videos, it remains unclear *which aspects of a social context* drive this elevated effect. Furthermore, differential theta power responses to social vs nonsocial live action might be related to *individual differences* in development.

Here, we applied NBO to study 6–12-month-old infants’ attention to a live actor. We aimed to identify the social cues that maximised frontal theta power for individual infants. To do this, we created a 2-dimensional stimulus space that varied by gaze direction and auditory content. Our first goal was to test the **feasibility of infant NBO in a naturalistic, live social context.** We hypothesised that infant EEG data attrition rate would be lower than the 25 % observed in traditional continuous EEG studies with 5- and 10-month-old infants involving video recordings (van der Velde and Junge, 2020b), given the greater variety of stimuli and the infant-guided stimulus selection and the live actor (Gui et al., 2022).

The second goal was to test **where optima were located** in the 2-dimensional stimulus space. In line with previous literature, we hypothesised that infants would prefer the most communicative condition, i.e. direct gaze and contingent infant-directed speech.

The third goal was to test the role of **individual differences** in parent-reported social behaviour for infants’ attention within a social context. We predicted infants with higher scores in social adaptive behaviour to be more likely to prefer behaviours that are directed towards the infant such as IDS/direct gaze. Further, because parental negative mood in relation to social settings was found to be associated with measures of infant social attention (Jones et al., 2017), we predicted infants of parents with more positive and less negative mood to be more likely to prefer the behaviours closer to the IDS/direct gaze condition in the stimulus space. Finally, *age* might affect infants’ attention in that either older infants show stronger attention towards IDS/direct gaze due to progressed social brain specialisation (Jones et al., 2015), or older infants show stronger attention towards averted gaze due to the emergence of joint attention skills around 9 months of age, allowing them to follow gaze to an external object (Mundy, 2018).

## 2 Materials and methods

### 2.1 Participants

Fifty-seven infants (20 females, 37 males) aged 6–12 months (M=262.60 days, SD=61.13 days) participated. The first n = 14 infants (3 females, 11 males; age in days M=298.12, SD=49.45) were tested with an algorithm tuned towards exploitation to assess the potential frequency of convergence (see section “2.4 Bayesian Optimization”); the following n = 43 infants (17 females, 26 males; age in days M=251.02, SD=60.56) were tested with an algorithm tuned to exploration to enable a more detailed mapping of the stimulus space. Informed consent was obtained from the parent prior to the study.

### 2.2 Stimuli and procedure

**Fig. 1:**
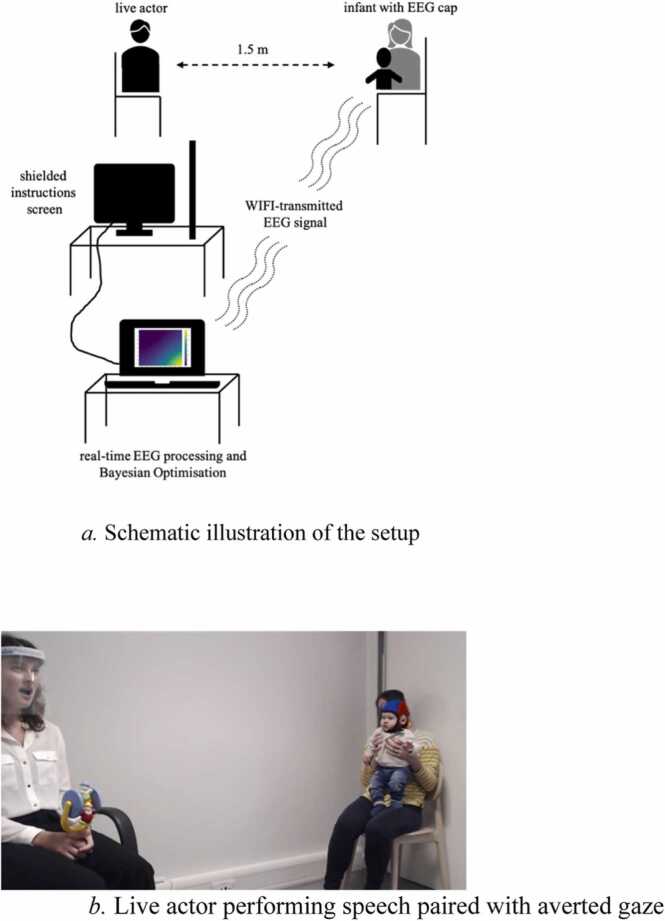
a. Schematic illustration of the setup. Fig. 1b. Live actor performing speech paired with averted gaze.

**Fig. 2:**
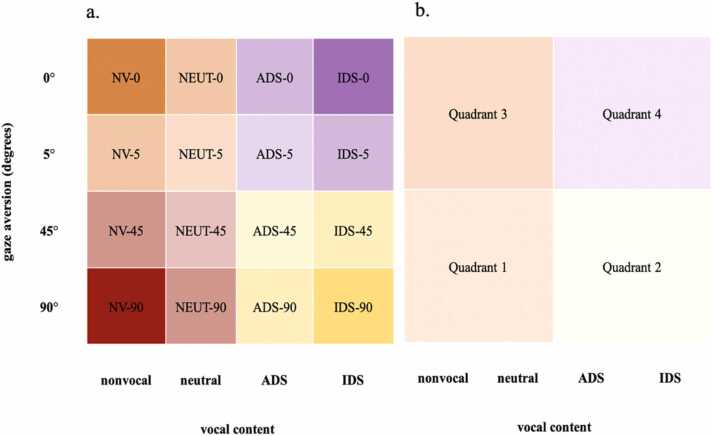
Stimulus space with stimuli varying across two dimensions (y-axis: the degrees of gaze aversion, x-axis: the infant-addressed vocal content). Colours represent analytical categories of stimuli, with (a.) four extremes of the space, (b.) being divided into quadrants.

Live stimuli were performed by a female adult seated 1.5 m opposite the child (Fig. 1). In total, seven live actors were involved (although each child only saw one actor), and performed the actions in an average of M = 7.50 sessions (SD=2.78, range=2–11). The child was seated on the parent’s lap, in a highchair, on the floor or held by the parent standing. Throughout the experiment, the actor sat with their body 45° averted from the child, displaying a mildly smiling facial expression. Stimuli varied across a 2-dimensional stimulus space by gaze direction (direct to 90-degrees-averted gaze) and auditory content (from infant-directed speech (IDS) over adult- directed speech (ADS) over neutral vocalisations to a non-vocal mechanical sound) (Fig. 2). Thus, each of these two dimensions included 4 steps, resulting in 16 possible stimulus combinations. The actors’ gaze was either 90°, 45°, 5°, or 0° averted. The head angle always varied along with gaze (Hains and Muir, 1996). Vocal content varied from nonvocal (operating a noisy toy), to vocal-neutral (harrumphing, yawning, coughing), to adult-directed speech (speaking the rhyme “The wheels on the bus” in normal speech), to infant-directed speech (speaking in infant-directed speech in a contingent, interactive way: “Hi NAME, how are you, NAME? Are you looking at me? Yes, you are looking at me!”). All actors were trained following a set procedure to increase consistency across actors.

Before the start of the experiment and in between stimulus presentation blocks, the actor was looking at a screen, with the head and gaze 90° averted from experimenter and child (baseline). Instructions for the actor were presented on that same screen using MATLAB Psychophysics Toolbox Extensions (Version 3) (Brainard, 1997, Kleiner et al., 2007). The screen was shielded by a black cover from the child’s view to minimise distraction. Each block consisted in the performance of one action. At the beginning of a block, a written instruction appeared on the screen for 3 s, followed by a bell ringing, indicating the start of the action. Each action lasted 8 s, with the end indicated by a second bell ringing. After the action episode, the actor looked back at the screen, while the recorded EEG signal for the block was being processed (∼ 6 s). During this time no stimuli were presented to offer the infant a break. If the infant was bored, the parent was allowed to naturally engage with the infant or the second experimenter handed a teething ring to them. When the instruction for the next block appeared on the screen, the stimulus presentation was resumed by the actor. If the infant was not looking, the parent or the second experimenter were redirecting the infant’s attention to the actor. The caregiver was asked to not interact with their infants during the interaction with the actor but were told they were welcome to interact with their infant during the breaks. In addition, caregivers were instructed to gently hold their child’s hands or let their child hold their hand if the infant tried to grab the cables.

Prior to the visit to the lab, parents filled in the Parent/Caregiver version of *Vineland Adaptive Behaviour Scale*s (Sparrow et al., 2005). We selected the “Interpersonal Relationships subdomain to reflect the child’s emerging social interaction abilities. In addition, parents completed the international short form of the *Positive and Negative Affect Scale* (Thompson, 2007), consisting of 10 adjectives reflecting either positive or negative affect. Parents were asked to rate the extent to which each adjective described how they generally felt on a 5-point Likert scale (1 = ”Very slightly or not at all”, 2 = “A little”, 3 = “Moderately”, 4 = “Quite a bit”, 5 = “Extremely”). Mean scores were derived for positive and negative affect separately, respectively.

### 2.3 Real-time EEG

EEG was recorded using the wireless gel based ENOBIO EEG system (NE Neuroelectrics, Barcelona, Spain). Data were transmitted to the acquisition software via WIFI connection. Within the 10–10 EEG coordinate system, 8 electrodes were used including 6 fronto-central electrodes of interest (Fz, FC1, FC2, C1, C2 and Cz) and two reference channels (P7 and P8; Figure 6.2). Frontocentral channels were chosen because previous studies reported a strong effect of elevated frontal theta power during social stimulations (Jones et al., 2015). The NE online reference electrodes (Driven Right Leg, DRL; the Common Mode Sense, CMS) were placed on the infants’ right mastoid using NE sticktrodes. EEG data was recorded in reference to the CMS channel and digitized at 500 Hz. Before starting the experiment, the signal quality was assessed using the NIC2 quality index including noise and offset of the signal *(Neuroelectrics User Manual, Part 3 NIC2, pp. 42–43)*. Additionally, the EEG signal was inspected visually. If the signal looked good and the quality index of most channels sufficient (green or orange), the experiment was started; otherwise, the cap fit was adjusted or additional gel applied in respective electrode holders.

After each block of live stimulus presentation, recorded EEG data were streamed to MATLAB and pre-processed automatically using custom MATLAB scripts (available upon request). The raw data from the block were detrended, demeaned and band-pass filtered (0.1–20 Hz). An 8-to-9-second window from stimulus onset to offset was segmented from the data and cut into 1-second epochs with 50 % overlap. Artefacts were identified using thresholding. For each epoch, data from a channel was excluded if amplitude exceeded +/−200 μV or an amplitude range of 400 μV, or if the range was < 0.0001 μV. Next, we calculated a reference signal per epoch by taking the mean across channels P7 and P8 (if artefact-free, otherwise the signal from the artifact-free channel only was taken). The reference signal was then subtracted from the artefact-free time series of each channel of interest (Fz, Cz, FC1, C1, FC2, C2). Here, we re-referenced the signals to the average of P7 and P8 to improve data quality and increase specificity to the selected frontal location.

After artefact-rejection and re-referencing, each artefact-free channel in each epoch was subjected to a Fast Fourier Transform with a Hanning window (hannwin function, MATLAB), and the power spectrum was calculated. The power spectrum was calculated as the absolute value of the Fourier coefficients for each sampled frequency (resolution of 1 Hz). The values were then multiplied by 2 and squared.

Next, another round of data quality checks followed: At each frequency in each epoch, a channel was excluded if the power value exceeded 3 times the standard deviation of the mean of the power values across all remaining channels in that sampled frequency in that epoch (as in (Jones et al., 2015)). Data were log-transformed to reduce skewness. At each sampled frequency, power values were averaged across epochs and then channels resulting in an overall power spectrum. We then divided power values in the 3 Hz, 4 Hz, 5 Hz and 6 Hz frequencies by the averaged power (1–35 Hz) to calculate relative power for the theta frequencies. Finally, we took the mean across the 3–6-Hz-frequencies to obtain relative theta power. This relative theta power was used as the key EEG metric for the NBO in the present experiment.

Sometimes lower frequency bands can be particularly contaminated by motion or sweat artifacts. We therefore ran additional off-line analyses to test whether the lower frequency bands had a substantiative influence on the relative theta values. We reasoned that if artifacts in the 1–2 Hz range had affected the signal, the correlation between relative theta values from the 1–35 Hz vs 2–35 Hz would be low. The results revealed a strong correlation (r = .74, p < .00001) suggesting that the influence of lower-frequency artifact is likely to be limited, though of course cannot be excluded.

In the final step of the real-time EEG analysis, the data quality of the block was evaluated and the algorithm decided how to proceed to the next block. The percentage of epochs that had survived the amplitude-based artefact rejection was calculated to ensure a sufficient level of data quality. Off-line data analyses after data collection was finished revealed that on average 78 % of the epochs were included per block (ranging between 15 % and 100 %, also see Table 1). During the sessions, after each block, if the percentage was equal to or higher than 15 % of all epochs across channels, the output was saved and passed on to the NBO to select the next live sampled behaviour from the stimulus space; otherwise, the block was repeated. Further, a bar chart displayed the number of epochs in each channel that had survived artefact rejection criteria and were included in calculating the signal, allowing the experimenter to identify channels with particularly few artefact-free epochs obtained in the current block and if needed to adjust or re-gel the electrodes to improve the EEG signal in those channels before continuing with the following block.

**Table 1:** Summary of epochs included across all blocks and infants assessed.

		**Mean**	**Standard deviation**	**minimum**	**maximum**
Total	Epochs included	100.15	31.92	18	136
	Epochs included (%)	77.56	24.16	15	100
Exploitative sampling	Epochs included	104.18	31.30	24	136
	Epochs included (%)	76.60	23.01	18	100
Exploratory sampling	Epochs included	98.35	32.08	18	136
	Epochs included (%)	77.99	24.68	15	100

### 2.4 Bayesian optimization

Neuroadaptive Bayesian Optimization (NBO) combines real-time analysis neurophysiological or neuroimaging data with machine learning in order to identify from a space of possible stimuli the one that elicits a pre-defined target brain state in an individual (Lorenz et al., 2017, Lorenz et al., 2018, Lorenz et al., 2016, da Costa et al., 2021). NBO uses a closed-loop design to model an unknown function (surrogate model) across a stimulus space and rapidly identify extrema in this model while only presenting a subset of stimuli. In each iteration of the loop, a stimulus from the space is being presented, and the response to it added to the model predicting the response function across the entire stimulus space. Here, the BO algorithm was programmed to identify which combination of gaze direction and vocal content elicited the strongest relative theta power in the individual infant from a range of different combinations of gaze directions and vocal content as presented in Fig. 2a.

The surrogate model, i.e. the model of the underlying function of how the brain response maps onto the stimulus space, is unknown and is iteratively built up over the course of the optimisation. We used Gaussian process regression with a Matern covarince kernel (Shahriari et al., 2015) to form the surrogate model; this approach can, in theory, approximate any function. An initial model is built after a burn-in phase with a few predefined stimuli before entering the optimisation phase. In this study, pre-defined burn-in stimuli were the four corners of the stimulus space (nonvocal / gaze 90 averted; nonvocal / direct gaze; IDS / gaze 90 averted; IDS / direct gaze, corresponding to NV-0, IDS-0, NV-90, IDS-90).

A pre-defined acquisition function selects the stimulus to present next, based on features of the surrogate model (Frazier, 2018). We chose the Expected Improvement (Shahriari et al., 2015) as the acquisition function, because it allows both exploration and exploitation within the optimisation process, enabling identification of the function extrema after a limited number of iterations.

The balance between exploitative and exploratory sampling of the space is defined by the *hyperparameter ξ* of the acquisition function. A lower ξ results in more exploitative sampling allowing more rapid identification of the stimulus eliciting the maximum in the unknown theta power function. A higher ξ results in more exploratory sampling allowing the algorithm to more extensively map out the unknown theta power function across the stimulus space, without aiming for rapid identification of the optimum stimulus. In this study, two different hyperparameters ξ were used. In the first part, we used exploitation sampling (n = 14) until we reached an attrition rate below 15 %, considerably lower than the 25 % observed in traditional continuous EEG studies with 5- and 10-month-old infants (van der Velde and Junge, 2020a). We then switched to exploration sampling (n = 43) to enable greater exploration of the space. In the *exploitation* sampling, we used an exploration/exploitation hyperparameter ξ value of 0.1. Pilot data revealed this value to be suitable to minimise the number of blocks needed to identify the optimum in the space eliciting the strongest theta power amplitude. In the *exploration* sampling, we used a hyperparameter ξ value of 1, as this would allow extensive exploration of the search space, in order to reveal how the individual’s brain response maps onto it. The exploitative sampling builds a stronger model of the maximum brain response, whereas the explorative sampling builds a better representation of the overall response across all stimuli.

The optimisation loop stops once a user-defined *stopping criterion* has been met. Here, this was defined to be reached when the same stimulus was sampled three consecutive times (as in (Lorenz et al., 2017)). If the stopping criterion was not established, the paradigm would stop after a maximum of 15 blocks (4 burn-ins + 11 optimisation iterations), in order to not exceed the infant attention span. In both cases, reaching the stopping criterion or the maximum of 15 blocks, the BO predicts the stimulus to elicit the strongest target response for the individual.

A more detailed description of the BO algorithm used in this study can be found in (da Costa et al., 2021).

### 2.5 Offline statistical analysis after data collection

The immediate outcome after a session was the position of the individual’s predicted optimum stimulus within the 2D-live stimulus space. Post-study group-level analyses were run on the distribution of the individual optima across the space, and how these related to measures of individual differences. The position of the individual optimum was operationalised in two ways: 1) as Euclidean distance from the most “social” condition (IDS/direct gaze) (“optimum-social distance”), with a shorter optimum-social distance reflecting that the predicted optimal stimulus was closer to the IDS/direct gaze stimulus in the 2D-stimulus space, and 2) the quadrant in which the optimum is located in the stimulus space. For the latter, the 4 × 4-stimulus space was divided in 4 parts, including the 4 behaviours around the respective corners (Fig. 2*a, b*). While only data from the exploitation sampling was used to evaluate the BO efficiency in reaching the pre-defined stopping criterion, data from both the exploitation and exploration sampling were collapsed for the remaining analyses. To do this, we made use of the fact that the algorithm updates the predicted model of the underlying function of brain response after each point of sampling, taking into account all samples collected of an infant up to that point.

To compare the NBO results with results obtained using a traditional approach, we conducted a repeated-measures ANOVA to test the difference in theta power between social (IDS/direct gaze, Quadrant 4) vs. nonsocial (Quadrant 1, nonvocal/averted gaze) conditions, including the number of blocks as covariate.

#### 2.5.1 Attrition rate and BO efficacy

To assess attrition, we calculated the proportion of infants who did not complete the paradigm, i.e. for whom the experiment was terminated before reaching either the stopping criterion or the maximum number of 15 blocks, for example due to poor data quality or the infant being tired or fussy. To assess efficacy of the BO algorithm, the proportion of infants reaching the stopping criterion was calculated among those completing the paradigm, as well as the average number of blocks needed.

#### 2.5.2 Distribution of optima in the 2D-live stimulus space

To test the prediction of infant natural pedagogy that most optima are located in the space quadrant including more ostensive cues (Quadrant 4), we calculated the proportion of optima per quadrant.

#### 2.5.3 Relation to age and social behaviour

Multinomial multiple logistic regressions were used to test whether the likelihood of converging in Quadrant 4 compared to the other Quadrants was associated with age (in days). We also tested the relationship between likelihood of converging in Quadrant 4 and social behaviour (*VABS “Interpersonal Relationships”* subdomain v-scale scores). Finally, we tested the relationship between likelihood of converging in Quadrant 4 and positive and negative parental affect (*PANAS Positive and Negative Affect Scale*). We expected optima in Quadrant 4 to be more likely in infants with higher scores on social behaviour measures, higher scores on the positive parental affect scale and lower scores on the negative parental affect scale, and in older infants.

## 3 Results and discussion

### 3.1 Success and efficiency in identifying individual infants’ optima

#### 3.1.1 Attrition rate and convergence

The first set of infants were tested with a low hyperparameter that favoured rapid identification of an optimum (exploitative hyperparameter). From the exploitation sample (n = 14), 2 infants (14 %) did not complete the study due to fussiness. This drop-out rate is favourable compared to typical screen-based paradigms (25 %, (Frazier, 2018)). Of the infants completing the study, n = 11 infants (92 %) reached the stopping criterion of the same stimulus in three consecutive blocks in the exploitation sample, after an average of 9 blocks (SD = 2.04, range: 6–12), while n = 1 infant (8 %) did not reach the stopping criterion and hence was presented with the maximum of 15 blocks. Reaching the stopping criterion is associated with the reliability of the measured responses as BO chooses where to sample in order to minimise the uncertainty of the function’s maximum position. Meeting the stopping criteria shows that the model is not changing the expected maximum response for three consecutive samples and the uncertainty of the maximum position is low. This shows that these hyperparameters can be selected to produce a reliable estimate at the level of the individual infant with a low drop-out rate. This approach may be particularly valuable for studies of individual differences in clinical groups, where high inclusion rates are paramount.

We then increased the hyperparameter estimate for the remainder of the sample to increase the amount of data collected per infant, and to allow us to map the full stimuli space. This was important to address our theoretical question about the role of gaze and vocal cues in social attention. From the exploration sample (n = 43), 18 infants (41.86 %) did not complete the paradigm. The reasons for drop-out were bad data quality (n = 5), an error in the script (n = 6), fussiness of the infant (n = 2), no output saved (n = 1) or a combination of these reasons (n = 4). On average, these infants completed m= 5.5 blocks (SD=4.08, range=1–13).

Besides experimental failures, this rate is higher than in the exploitation sample because infants in the exploration sample were less likely to reach the stopping criterion, as it was designed for the exploitative phase, and therefore the duration of the task was extended, resulting in decreasing data quality and increasing fussiness. Only one of the 25 infants in the exploration subsample (4 %) reached the stopping criterion (after seven blocks), while all other infants (n = 24) were presented with the maximum of 15 blocks, as expected given the algorithm was tuned towards greater exploration of the space in this subsample. Overall, this indicates that mapping a broader range of the stimulus space will exert a cost in attrition rates; increasing the speed of inter-trial intervals with more powerful computational approaches may be helpful.

### 3.2 Distribution of individual optima across the 2D-live stimulus space

**Fig. 3:**
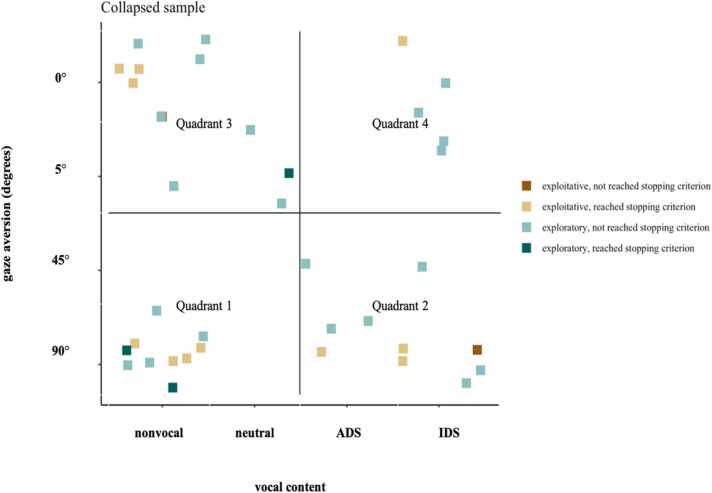
Individual optima across the 2-dimensional live social experiences space of the collapsed sample of infants who completed the paradigm, i.e., either reached the early stopping criterion or the maximum of 15 blocks (n = 37). Quadrant 1: n = 10 (27 %); Quadrant 2: n = 10 (27 %); Quadrant 3: n = 12 (32 %); Quadrant 4: n = 5 (14 %).

**Fig. 4:**
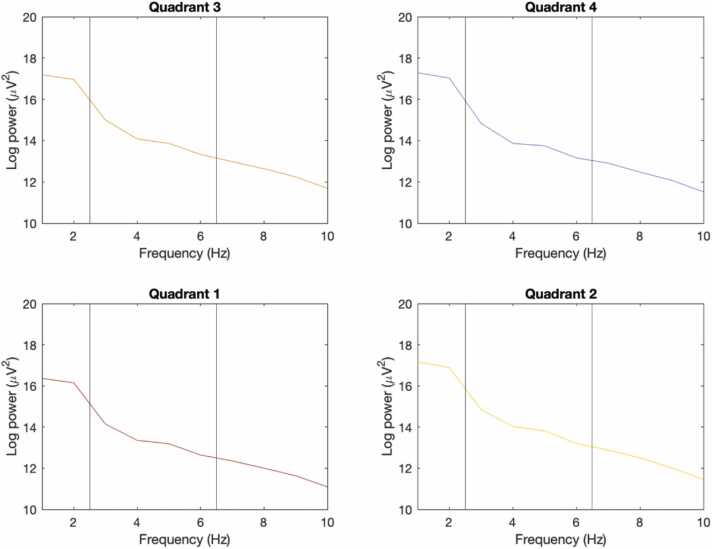
Mean log transformed power spectra for each quadrant.

The distribution of individual optima across the stimulus space is visualised in Fig. 3. Of note, optima are calculated for any infant who either reached the stopping criterion or completed all 15 blocks; analyses were collapsed across the exploration and exploitation samples. Fig. 4 displays the log transformed power spectra averaged across infants for each of the 4 quadrants.

The *proportion of optima per quadrant* of the 37 infants who completed the paradigm did not differ significantly between quadrants, though numerically *fewer* infants preferred speech with direct gaze (4-sample test for equality of proportions: χ² (3) = 3.86, p = .2). Similarly, the *proportion of optima per corner* of the space did not differ significantly between corners (4-sample test for equality of proportions: χ² (3) = 4.85, p = .1).

Power spectra were averaged across all available blocks for each quadrant and across infants. We visualised power spectra across the lower frequencies here due to our focus on the theta frequency band (3–6 Hz, between the vertical lines).

This pattern of findings shown in Fig. 3 suggests that infant attention was not generally strongest during behaviours that were most directed towards the infant as suggested by natural pedagogy theory. Instead, when looking at individual optima, there was no overall difference in theta power for any of the Quadrants. Although unexpected based on the natural pedagogy theory, these results are in line with recent reports using naturalistic paradigms. For example, Angelini and colleagues found that theta power was higher during incongruent gaze shifts towards an object during a naturalistic paradigm (Angelini et al., 2023). Further, Marriot Haresign and colleagues investigated brain synchronisation during parent-child live interaction episodes and found that gaze shifts towards the child did not increase their theta power nor parent-child inter-brain synchrony (Marriott Haresign et al., 2022). It might be that in less structured contexts, that better represent the complexity of everyday interactions, inter-individual variability in brain responses might be enhanced compared to those emerging in highly controlled screen-based paradigms. This highlights the importance of individualised designs that go beyond traditional designs that compare a subset of pre-defined stimuli. Novel approaches that integrate multiple theoretical accounts by using multi-dimensional experimental design spaces have the potential to generate reliable theoretical knowledge about the social brain development in complex, real-live contexts (Almaatouq et al., 2022).

### 3.3 Relation between individual optima and measures of behaviour and age

Multiple multinomial logistic regression of the likelihood of converging in Quadrant 4 (speech/direct gaze) compared to the other quadrants did not reveal significant changes with age (all ps >.1). Thus, infants did not become more likely to show stronger attention for infant-directed speech and direct gaze with age. However, infants with stronger social skills (higher VABS Interpersonal Relationships subdomain scores) showed an increased likelihood of converging in Quadrant 1 (non-speech/averted gaze; log =.55, p = .048, z = 1.971) compared to Quadrant 4 (speech/direct gaze).

This is broadly consistent with the results of a repeated-measures ANOVA on theta power computed traditionally within each half of the space defined diagonally (see SM) showing significantly stronger relative theta power in the nonsocial vs. social condition (F (1, 35) = 10.28, p = .003 ηp^2^ = .23); further, the number of blocks administered to infants was higher for Quadrant 1 (non-speech/averted gaze) vs 4 (speech/direct gaze), indicating the algorithm had more often identified optima in that location (F (1, 35) = 9.14, p = .005, ηp^2^ = .21). Thus, relative theta power was higher in the non-speech/averted gaze condition than the speech/direct gaze condition (Table 2); the algorithm sampled there more frequently; and was more likely to converge or show an optimum there for infants with stronger social skills.

**Table 2:** Relative theta power per quadrant and corner of the stimulus space (in μV^2^).

			**Quadrant 1 (nonspeech/ averted gaze)**	**Quadrant 2 (speech/averted gaze)**	**Quadrant 3 (nonspeech/ direct gaze)**	**Quadrant 4 (speech/direct gaze)**
**Relative theta power**	*Overall quadrant*	*Mean (SD)*	1.86(1.74)	2.22(0.96)	2.10(0.70)	1.74(3.33)
	*Corner only*	*Mean (SD)*	1.78(1.97)	2.07(0.72)	2.04(0.89)	1.23(4.59)

These findings are again not in line with the natural pedagogy theory, which would predict stronger attention during the infant-directed speech/direct gaze condition. Previous EEG work has provided some support for elements of natural pedagogy. For example, live or video-based episodes of women singing with direct gaze for blocks of around a minute have previously been shown to elicit stronger theta power in comparison to blocks of nonsocial stimuli (e.g. toys moving; (Haartsen et al., 2022, Jones et al., 2015, van der Velde et al., 2019). These findings may differ from the present study because of the block design, meaning an extended experience of one type; indeed, theta power increases during each block (Jones et al., 2020). Further, the use of singing is associated with greater entrainment of theta signals, which may be diminished or absent for briefer epochs of speech (Leong et al., 2017b). Alternatively, in the present study the nonspeech/gaze averted condition could have been interpreted by the infant as representing a joint attention (JA) situation (even though the actor was not looking at the toy making the noise, but at a third point). Infants start to be engaged with objects that are looked at by a social interaction partner towards the end of the first year of age (Mundy, 2018). The ability to follow another person’s gaze, the basis for engaging in Joint Attention, has been suggested to develop by 6 months of age (Gredebäck et al., 2010) and signs of JA have been observed during a naturalistic paradigm in infants from 9 months of age (Cleveland and Striano, 2007). Previous studies on JA showed that infant theta power differs between conditions using gaze cues towards or away from objects (Angelini et al., 2023, Michel et al., 2015) suggesting that in the present study infants who already started acquiring this skill were more attentive to the nonspeech/gaze averted condition than with others. The context of the experiment -in which infants experienced some direct gaze with infant-directed speech mixed with other epochs with averted gaze -may have also increased the likelihood that broader interpretations of the social context as a whole influenced responses to individual stimuli. Although these naturalistic aspects of the paradigm make interpretation more difficult, they are also clearly present in real-world social interactions. Our findings may thus be consistent with a broader range of research in which modulation of infant brain activity in response to distinct cues may differ when they are presented in isolation to when they are experienced embedded in a naturalistic setting (Angelini et al., 2023, McDonald and Perdue, 2018, Hoehl et al., 2014, Lachat et al., 2012).

We also examined whether infants of caregivers experiencing lower positive affect were more likely to prefer actions characterised by nonvocal/direct gaze or IDS/gaze averted vs. IDS/direct gaze. We showed that an increased likelihood of converging in Quadrant 2 (speech/averted gaze; log = −0.482, p = .037, z = −2.083) or Quadrant 3 (non-speech/direct gaze; log = −.462, p = .034, z = −2.13) compared to Quadrant 4 (speech/direct gaze) was significantly related to lower scores on the PANAS Positive Affect subscale. Previous research suggested that parental social motivation (Jones et al., 2017) and maternal stress (Pierce et al., 2021) have previously been found to associate with infant theta power. Possibly, our results are in line with the idea of a mutually reinforcing mechanism in early parent-child interaction (Shultz et al., 2018). Indeed, parental engagement in social exchanges (Wass et al., 2018) and infant-directed actions (Meyer et al., 2023) have been shown to modulate theta power in the infant. This individualised approach applied during live interactions in clinical populations (for example, with mothers who suffer post-natal depression, or children with emerging difficulties in social interactions) might have the potential to identify which interactive behaviours capture an individual’s attention the most and be used to support early parent-child relationships.

### 3.4 Limitations

NBO requires pre-specification of all aspects of the EEG analytic pipeline, thus building in elements of preregistration to the paradigm. This substantially reduces analytic flexibility, which may in turn increase the likelihood of ‘null’ findings at the group level. Future work should examine whether our results are influenced by the parameters selected to control the algorithm. For example, it is possible that defining different criteria for convergence would have yielded different results. Additionally, analyses were collapsed across exploration and exploitation samples to maximise power. This first application of NBO during a real-time EEG naturalistic social paradigm in infancy indicated that a more exploitative sampling is preferable as it reduces attrition rate in developmental populations.

A further limitation of this study shared with other naturalistic studies is the non-standardisation of acted out behaviours. Despite training and scripts for the behaviours, it is possible that naturalistic dynamics that could not be pre-scripted without compromising the validity of the behaviour influenced the infants’ responses. For example, during the IDS / direct-gaze condition, actors were responding contingently to the infant’s behaviour to enact spontaneous naturalistic infant-directed behaviour that infants experience in everyday life, and hence the exact choice of words used could differ between infants.

Epochs containing artefacts (defined as amplitude exceeding +/−200 μV, amplitude range exceeding 400 μV, range consistently below < 0.0001 μV) were excluded from the further analysis. Further techniques like Independent Component Analysis were not considered to provide robust results with the amount of data obtained in a 8-s-block. Despite our artifact-detection approach, we recognise there may be some remaining eye movement, muscle artifact or low-frequency artifact in the EEG signal.

Finally, the present real-time study purposefully did not control infants’ continued looking at the stimulus, to a) not compromise the naturalistic character of the study, and b) not exclude part of the measure of interest (i.e. social attention) by discarding the segments during which the infants were not looking at the experimenter, likely reflecting attentional disengagement. However, this again makes interpretation more difficult. Of note, video-coded looking times on a subset of 12 infants (see Supplementary Materials and Table S1) were not significantly associated with theta power within each quadrant in a linear mixed model accounting for the random effect of individual infants (log likelihood = 7.82, AIC = −3.64, BIC=7.07, effect of look = 0.17, SE=0.53, t(33) = 0.33, p = 0.74; effect of quadrant = −0.0097, SE= 0.14, t(33) = −0.07, p = 0.94; look by quadrant interaction= 0.024, SE= 0.17, t(33) = 0.14, p = 0.89), indicating that behavioural attention is unlikely to have a confounding effect on the results.

### 3.5 Future directions

The present study suggests that NBO is a viable alternative method for probing infant brain responses at the individual level. Further, tuning the algorithm towards exploitation was associated with low drop-out rates, increasing the viability of the method. The high degree of convergence within the exploitation sample indicates that the number of trials and data quality recorded yielded reliable estimates of relative theta power at the level of the individual infant; high reliability is critical in developing individualised biomarkers and may have utility in the study of infants with emerging neurodevelopmental conditions like autism.

NBO may provide a useful tool in which to explore further aspects of live social contexts, such as contingency in adult responses to infant behaviour. Some theorists suggest that contingency is a key feature triggering infants’ attention (e.g., (Murray and Trevarthen, 1986)). However, there is evidence that the level of contingency experienced in daily interaction with their caregiver determines infants’ preferred level of contingency (Bigelow, 1998). This suggests an individualised approach such as NBO would be ideal to investigate the role of contingency in social attention. Further, taking into account the temporal dynamics of social interaction might be important in investigating patterns underlying individual attention patterns, such as whether the behaviour presented in the previous block influenced responses to the present block.

Future NBO studies should also consider combining different neural metrics. These could include further neural measures that have been shown to reflect social attention engagement in the first year of life (e.g., theta connectivity; (van der Velde et al., 2021)), as well as components of infant behaviour, such as smiling, which has been suggested to be a good indicator for social attention engagement in very young infants (Muir and Hains, 1993). The more differentiating and reliable the (combined) target neural metric, the fewer iterations are needed for converging to the predicted optimum and the higher the success of the NBO experiment.

## 4 Conclusions

This study used NBO with infant EEG to test how variation in social cues within a naturalistic context influenced the neural correlates of attention in typically developing infants. The optimization algorithm identified the action that most likely maximised brain activation for most infants who participated in the paradigm in which rapid convergence was aimed for, showing that the method produced a robust signal reliably differentiating between different points in the stimulus space. No overall enhanced attention for specific combinations of gaze direction and vocal content was observed, challenging the generalisability of some theories of early social interaction. However, attention depended on the infants’ social behaviour skills and parental mood, suggesting that individual differences play a role in which aspect of social interaction is experienced as most attention-capturing. Taken together, new experimental approaches like NBO can yield robust measurement of infant brain function in a naturalistic social context and could help accelerate the discovery of meaningful individual differences in infant brain function.

## Declaration of Competing Interest

The authors declare that they have no known competing financial interests or personal relationships that could have appeared to influence the work reported in this paper.

## Data Availability

Data will be made available on request.
