# Supplementary material for: Real-time monitoring of infant theta power during naturalistic social experiences
Source: Dev Cogn Neurosci. 2023 Sep 21;63:101300. doi: 10.1016/j.dcn.2023.101300 (PMC10523417; doi:10.1016/j.dcn.2023.101300)
Supplement: Supplementary file 1 — Supplementary material. [file mmc1.docx]

**Supplementary Material**

*Additional analyses*

To compare the individual optima produced by the BO approach with traditional analysis of relative theta power across the group, ANOVA was used to test whether relative theta power (dependent variable) differed by quadrant (4 levels), with social behaviour, parental affect and age as covariates.

To test the distribution of group-level relative theta power across the stimulus space, an ANOVA of relative theta power by quadrant revealed no significant effect (F (3, 108) = 2.41, p = .07, ηp^2^ = .06). As with the individual optima, we calculated an additional ANOVA comparing relative theta power across only the *corners* of the space. This analysis revealed a significant effect of Corner on relative theta power (F (3, 108) = 3.14, p = .028, ηp^2^ = .08), with relative theta power being greatest over infant directed speech with averted gaze and nonverbal with direct gaze (Quadrant 3 and 2).

To establish whether the 2D stimulus space reflected a gradient from not socially engaging to socially engaging, spread across two dimensions of social interaction (gaze direction and vocalisation), a repeated-measures ANOVA of relative theta power by stimulus-space-half (socially more engaging half vs. socially less engaging half) was calculated including all values obtained for stimuli from one of the two halves, while ignoring the values obtained for stimuli on the midline of the space (*Figure S1*). The number of blocks contributing to each half per child was included as covariate. We expected greater relative theta power for stimuli in the socially engaging half, based on previous findings (e.g., Jones, Venema, Lowy, Earl, & Webb, 2015)*.* Relative theta power values were transformed to reduce skew and the influence of outliers (transformation method recommended by R *bestNormalize,* Peterson, 2021: orderNorm).


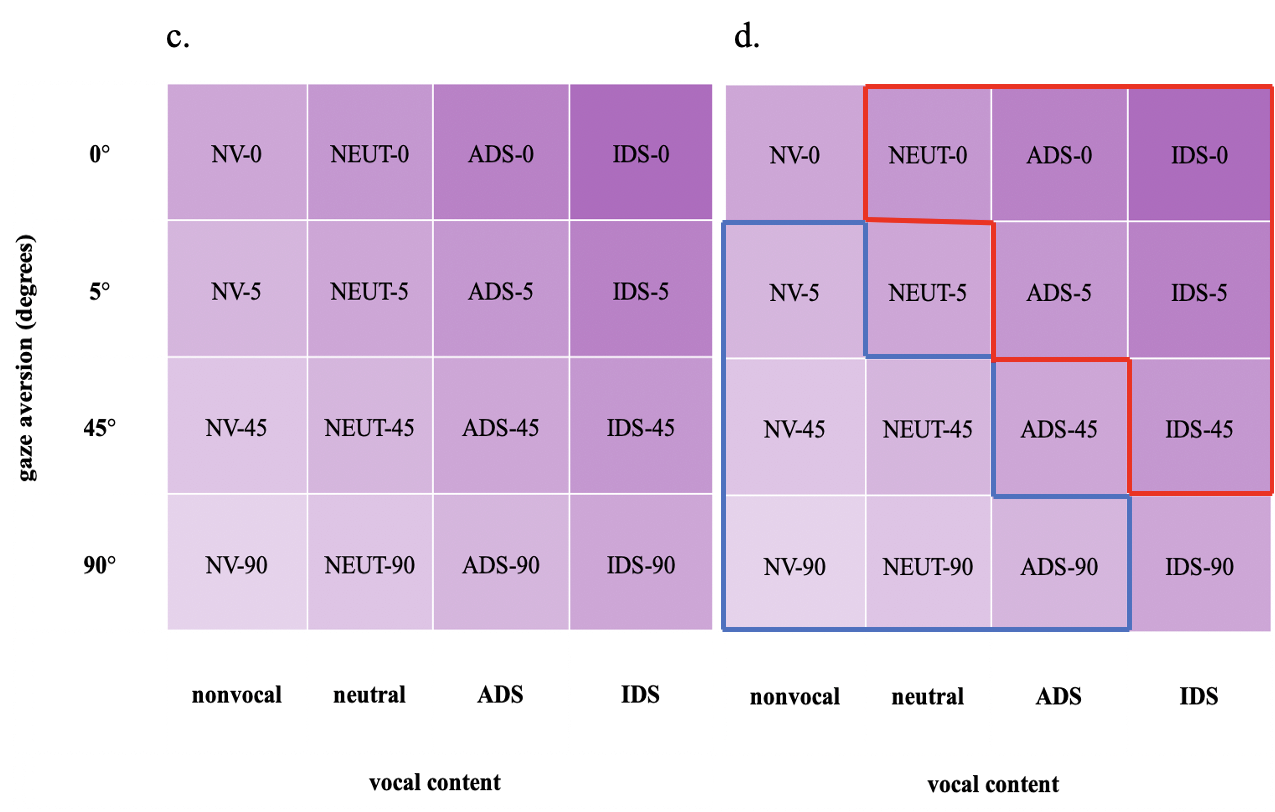


*Figure S1*. Stimulus space with live actions ranging from non-social (NV-90) to social (IDS-0) (left), and a proposedly less socially engaging half (blue) and a more socially engaging half (red) (right).

*Video-coding procedure*

Video recordings of the testing session of a subset of 12 infants were coded using ELAN (v6.3) to obtain looking times at the experimental action. The video recordings were chosen among the testing sessions of children in the exploration sample, because they generally included more blocks.

Microanalytic coding on 1-second units was performed by a researcher blind to the scope of the study. The coder initially annotated which of the 16 stimuli was presented marking the start and end time of each block based on the instructions presented on the experimenter’s screen. Within the block segments, the coder annotated the units when the child’s face and/or gaze were directed towards the actor or the toy.

For each infant, the sequence of coded stimuli was compared with the outcome of the Neuroadaptive Bayesian Optimization (NBO) to make sure they were consistent. Whenever a block was repeated due to data quality issues (see Methods), only the looking time to the last of the repeated blocks was retained, in line with the approach used for the NBO.

The proportion of looking time to each stimulus for each infant was obtained by dividing the total duration of the segments when the child was looking at the stimulus by the total duration of the block. These were averaged across stimuli within each quadrant and across infants, as shown in Table S1.

Additionally, we used a linear mixed model of the form theta ~ look duration*quadrant, random = list(~1| ID) to test the association between theta power and looking time across quadrants and infants (data shown in Figure S2).

*Table* *S1*. Proportion of looking time to the stimuli in each of the four quadrants of the stimulus space for 12 infants.

|  |  | | **Quadrant 1**  **(nonspeech/ averted gaze)** | | **Quadrant 2**  **(speech/**  **averted gaze)** | | **Quadrant 3 (nonspeech/ direct gaze)** | | **Quadrant 4**  **(speech/**  **direct gaze)** | |  |
| --- | --- | --- | --- | --- | --- | --- | --- | --- | --- | --- | --- |
| **Proportion of looking time** | | *Mean (SD)* | | 0.83 (0.09) | | 0.71 (0.08) | | 0.79 (0.15) | | 0.76 (0.06) | |


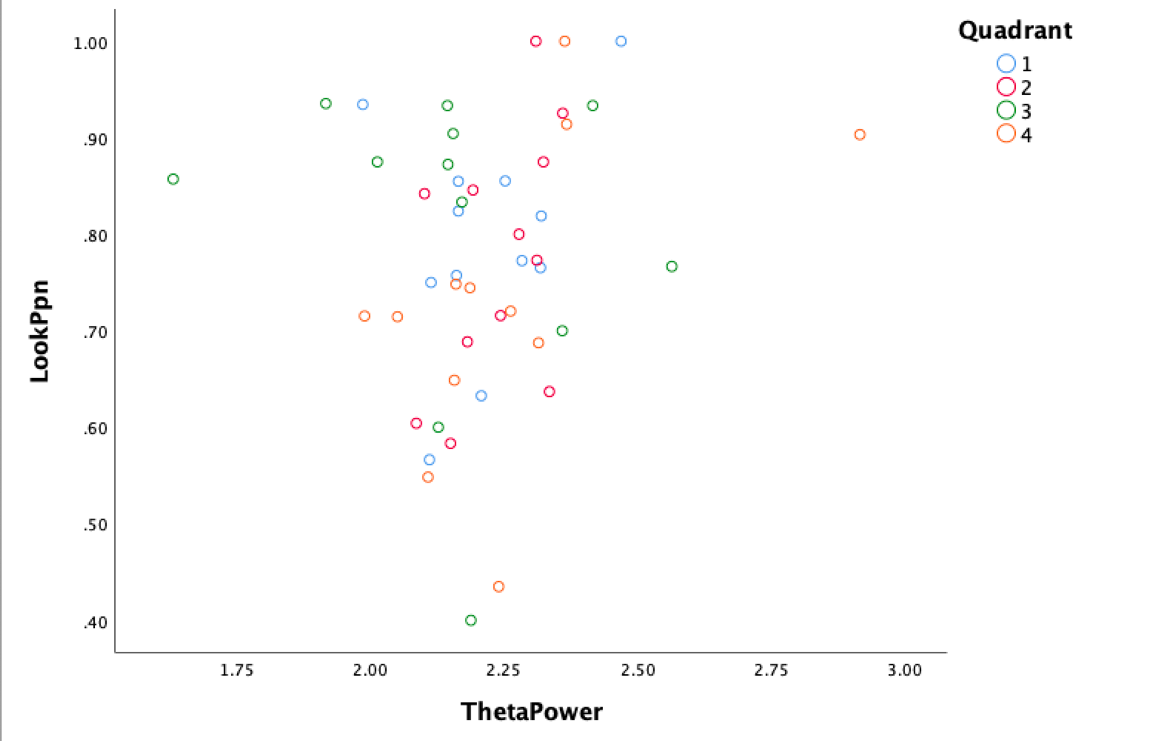


*Figure S2*: Lack of association between theta power and looking time per quadrant.
